# Clinical and Cardiovascular Magnetic Resonance Imaging Features of Cardiac Amyloidosis

**DOI:** 10.31083/j.rcm2410291

**Published:** 2023-10-16

**Authors:** Marco Tana, Claudio Tana, Alessandro Panarese, Cesare Mantini, Fabrizio Ricci, Ettore Porreca

**Affiliations:** ^1^Internal Medicine and Cardiovascular Ultrasound Unit, Medical Department, SS. Annunziata Hospital, 66100 Chieti, Italy; ^2^Department of Neuroscience, Imaging and Clinical Sciences, “G. D'Annunzio" University of Chieti-Pescara, 66100 Chieti, Italy; ^3^Geriatrics Clinic, SS. Annunziata Hospital, 66100 Chieti, Italy

**Keywords:** cardiac amyloidosis, diagnosis, magnetic resonance imaging, strain

## Abstract

Amyloidosis is a systemic disease characterized by the accumulation of insoluble aggregates in various organs, leading to parenchymal damage. When these amyloid fibrils are deposited in the extracellular matrix of the cardiac structures, the condition is referred to as cardiac amyloidosis (CA). The extent of organ involvement determines the degree of cardiac impairment, which can significantly impact prognosis. The two most implicated proteins in CA are transthyretin and misfolded monoclonal immunoglobulin light chains. These proteins give rise to two distinct clinical forms of CA: transthyretin amyloidosis (ATTR-CA) and light-chain amyloidosis (AL-CA). ATTR-CA is further classified into two subtypes: ATTRm-CA, which occurs at a younger age and is caused by hereditary misfolded mutated proteins, and ATTRwt-CA, which is an acquired wild-type form more commonly observed in older adults, referred to as senile amyloidosis. While AL-CA was considered the most prevalent form for many years, recent autopsy studies have revealed an increase in cases of ATTRwt-CA. This narrative review aims to describe the clinical and imaging features of CA, with a particular focus on cardiac complications and mortality associated with the AL form. Early identification and differentiation of CA from other disorders are crucial, given the higher risk and severity of cardiac involvement in AL-CA. Furthermore, emphasis is placed on the potential utility of cardiovascular magnetic resonance imaging in detecting early cases of CA.

## 1. Introduction

Amyloidosis is a systemic disease caused by low molecular weight protein accumulation in the extracellular area, causing different degrees of parenchymal damage [1]. The condition is defined as cardiac amyloidosis (CA) when the amyloid fibrils are deposited into the extracellular space of the heart, leading to cardiac impairment and poor prognosis [2].

More than 30 fibril-forming proteins have been found [3], although the most frequent are represented by transthyretin and misfolded monoclonal immunoglobulin light chains, which lead to two different clinical forms called transthyretin amyloidosis (ATTR) and light-chain amyloidosis (AL). The two types of cardiac involvement are defined with the acronyms ATTR-CA and AL-CA, respectively [2, 4, 5].

There are two types of thransthyretin, a hereditary misfolded mutated protein, which is associated with ATTRm-CA and occurs in younger age, and an acquired wild-type, which is called ATTRwt-CA and occurs most often in the elderly (senile systemic amyloidosis) [4, 5].

Complete information about the epidemiology of CA is still unknown. It has been hypothesized that the heart involvement from amyloidosis is underestimated in clinical studies, being more detected from autopsy exams [5], especially for the senile form ATTRwt-CA [6].

A study conducted between 2002 and 2012 found a total of 4746 and 15,737 incident and prevalent cases of CA in 2012, respectively, and a significant prevalence rate increase from 2000 to 2012, with a total count of 8 to 17 per 100,000 person-years and incidence rate of 18 to 55 per 100,000 person-years. The incidence and prevalence have increased after the year 2006, suggesting the occurrence of an improvement in diagnostic techniques in the last decades [7].

CA seems to be especially frequent among Black patients and men ≥65 years, and prevalence increases with age [4]; therefore, these patients should be routinely investigated when they are hospitalized with a new diagnosis of heart failure [7, 8].

A high clinical awareness is, therefore, essential because an early diagnosis could be useful for a correct treatment and could change the patient outcome. The median survival time in untreated vs. treated patients is 9 to 24 months for AL-CA and 7 to 10 years for ATTR-CA, respectively [4]. Cardiac magnetic resonance imaging (CMRI) makes a significant contribution in terms of early identification of cardiac involvement in amyloidosis. The existing body of literature has extensively investigated the diagnostic and prognostic implications of CMRI. However, there is still a knowledge gap that needs to be addressed. Therefore, the primary objective of this review is to bridge this gap by providing a more comprehensive understanding of the utility of CMRI in the setting of suspected CA. In addition to this, the review will also provide a global perspective on the pathophysiological and clinical features of CA. By consolidating the available research and presenting new insights, this review aims to enhance our understanding of the role of CMRI in CA and provide valuable information for clinicians and researchers in the field.

## 2. Pathologic Findings and Clinical Features 

In CA, amyloid fibrils are composed of insoluble fibers that are resistant to degradation, and they are deposited in the extracellular matrix of the heart. The accumulation is associated with ventricular wall thickening, rigidity, diastolic dysfunction, increased filling pressures, and progressive development of heart failure, which is typically associated with preserved ejection fraction (HF-PEF) [9]. Cardiac involvement often spares the apical area, while the basal zone is more often affected (‘apical sparing’). The underlying mechanisms are unknown, but some authors have hypothesized a lower deposition of amyloid fibrils in the apical site, the occurrence of segmental basal apoptosis due to increased wall stress, or a different myocites deposition [10].

The mean impairment of thickening starts from the subendocardial layer at the basal zone of both ventricles. Then, it continues with a transmural trend in the medial portions, sparing the apical areas up to an advanced stage of the disease (‘apical sparing’) and results in decreased systolic contractility and diastolic impairment [11].

The extracellular deposition of amyloid fibrils can change the normal atrioventricular (AV) conduction and favor the occurrence of re-entry ventricular and atrial arrhythmias, such as atrial fibrillation (AF) [12]. AF can also be associated with atrial dilatation due to elevated filling pressure [13]. An isolated involvement of one or both atria, defined as isolated atrial amyloidosis, has been reported in the literature and can be associated with AF or thromboembolic events, including cerebral stroke without ventricular involvement or systemic disease [14]. In addition to amyloid fibril deposition, some authors have hypothesized a local hyperproduction of atrial natriuretic peptide (ANP) [13, 15].

Other heart components can be affected by deposits of amyloid fibrils, such as the AV or semilunar valves, resulting in different degrees of stenosis [16]. Also, extracardiac structures such as pulmonary vessels can be involved, causing pulmonary arterial hypertension [17].

The right heart may also be involved, either by isolated or global amyloid accumulation or by a direct rising of the left ventricular (LV) filling pressure, which in turn increases the pulmonary vascular resistance, and pulmonary arterial pressure and favors a chronic right ventricular (RV) afterload and RV remodeling (hypertrophy in the early and dilatation in a late phase of disease), leading to a final reduction of RV contractility [17].

Tricuspid valve regurgitation (TR) is a common finding of CA, and it is an expression usually of amyloid fibril accumulation, or it is secondary to RV dilatation and papillary muscle displacement due to chronic increase of LV filling pressure [18].

Another possible complication is the occurrence of major adverse cardiac events (MACE), especially myocardial ischemia, if amyloid fibrils are deposited into the coronary vessels with vascular occlusion [19].

## 3. Diagnosis of CA by a Multimodality Imaging Approach, the Role of Cardiac Magnetic Resonance Imaging (CMRI) 

Diagnosis of CA is really challenging because symptoms and signs are usually non-specific, and CA can be misdiagnosed with other conditions such as aortic stenosis or heart failure. A recent European Society of Cardiology (ESC) consensus paper raises the suspect of CA in case of ventricular wall thickness ≥12 mm in association with red flags such as sensory and autonomic dysfunction, biceps tendon rupture, skin bruising, proteinuria, peripheral polyneuropathy, bilateral carpal tunnel syndrome, hypo or normotension if previously hypertensive, or electrocardiographic changes such as AV conduction disturbances, pseudo Q waves or decreased QRS voltage to mass ratio, and/or CMRI findings such as late gadolinium enhancement (LGE) or echocardiographic reduction of longitudinal strain with apical sparing [2].

A final diagnosis of CA can be obtained with two strategies: an invasive approach, based on endomyocardial biopsy or extracardiac biopsy positive for amyloid associated with echocardiographic and/or CMRI findings, or with a non-invasive approach (only for ATTR-CA) which needs the combination of a grade 2 or 3 cardiac uptake at diphosphonate scintigraphy, negative serum and urine immunofixation, negative serum free light chains and the occurrence of echocardiographic and/or CMRI criteria. Grade 2 or 3 bone scintigraphy has a specificity of approximately 95–100%, also without the presence of serum and urine monoclonal components [2]. Another nuclear imaging method for ATTR evaluation is Single Photon Emission Computed Tomography (SPECT), and radionuclide tracers for amyloidosis also include amyloid-directed molecules and positron emission tomography (PET) amyloid agents, but their use in clinical practice is limited by their low availability [20, 21].

Genetic testing is indicated in patients with a diagnosis of ATTR-CA in order to distinguish between ATTRwt and ATTRm forms and direct management with novel specific therapies, and to guide familial screening [2].

While endomyocardial biopsy is considered the gold standard method for establishing a definitive diagnosis [2], its routine use in clinical practice is limited due to its complexity, sampling error, and the requirement for skilled operators. Additionally, it is not without potential peri-procedural complications [22]. The recent improvement in the quality of diagnostic imaging and the introduction of more advanced software techniques has led to a significant improvement in non-invasive diagnosis of CA [8, 22].

Echocardiography is the first tool for assessing a suspected case of CA. It easily screens patients at the bedside without any risk of radiation exposure and does not need any contrast agent injection. Most typical echo features are reported in Table 1 (Ref. [2, 11, 23, 24, 25, 26, 27, 28, 29, 30, 31, 32, 33, 34, 35]) [36]. CMRI, in combination with a biopsy of extracardiac tissue, represents the gold standard for the diagnosis of CA and is a good alternative to endomyocardial biopsy when this is contraindicated or not available [2].

**Table 1.** **Main echocardiographic and cardiac magnetic resonance (CMR) findings in CA**.

Echo [2, 11, 27]	Strain Echo imaging [27]	CMR [23, 24, 25, 26, 27, 28]	Strain CMR imaging [25, 26, 27, 28, 29, 30, 31, 32, 33, 34, 35]
Ventricle hypertrophy	Reduced LVS, especially in the basal level with apical sparing (“bull’s eye” sign)	Increased LV thickness	Reduced GVS (circumferential, radial, longitudinal), especially in the basal level with apical sparing
Atrial walls thickening	Reduced AS	Atrial walls thickening	Reduced reservoir, conduct, and booster AS and reduced ASR
Valves infiltration with variable regurgitation and sclerosis		Subendocardial LGE (“zebra-pattern”) with non-coronary distribution	
		Transmural LGE with non-coronary distribution	
		Increased T1 mapping values (influenced by the field strength, pulse sequence, and cardiac phase) and ECV values (≥40%)	
Restrictive configuration: large atrial and small ventricles, diastolic impairment		Restrictive appearance (dilated atria and small ventricles)	
Systolic dysfunction in later stages		Reduced EF	

CMR, cardiac magnetic resonance; LVS, longitudinal ventricular strain; LV, left ventricular; GVS, global ventricular strain; AS, atrial strain; ASR, atrial strain rate; LGE, late gadolinium enhancement; ECV, extracellular volume; EF, ejection fraction; CA, cardiac amyloidosis.

### 3.1 CMRI: Classic Features

CMRI is a powerful, non-invasive imaging technique that can provide detailed images of the heart’s structure and function without using radiation. MRI can be a valuable tool for diagnosis, assessment, and monitoring of the CA.

CMRI is able to show morphological (thickness, effusion), functional (ejection fraction (EF), strain), and tissue (LGE and T1 mapping and extracellular volume (ECV)) findings of CA.

The late gadolinium enhancement (LGE) technique allows to identify areas of expanded extracellular space due to the deposition of abnormal amyloid protein and/or ischemic fibrosis due to capillary obstruction by fibrillar deposits [8]. This information is crucial for diagnosis and understanding the extent of disease involvement, but it requires intravenous injection of gadolinium-based contrast medium during a CMRI scan performed at least 10 minutes after the injection to obtain optimal contrast between the normal myocardium and damaged tissue [2, 8]. The LGE pattern is diffuse and predominantly sub-endocardial, unlike infarction and other cardiomyopathies [8]. CMRI has demonstrated high accuracy in detecting cases of CA, and the widespread diffusion of native T1 and ECV mapping has allowed the early identification of both types of amyloidosis [37].

T1 relaxation time describes how quickly the longitudinal magnetization of a tissue returns to its equilibrium after being perturbed by a radiofrequency pulse. T1 mapping involves acquiring a series of images in multiple heartbeats using different inversion times to measure the T1 relaxation time in various tissues. This technique can help differentiate between healthy and diseased tissues, as a result of different T1 relaxation times [38].

The most common T1 mapping technique is the Modified Look-Locker Inversion recovery (MOLLI) pulse sequence, which enables the determination of T1 times in a single breath hold over 17 subsequent heartbeats [39]. The Shortened MOLLI (ShMOLLI) technique makes use of sequential inversion-recovery measurements with only 9 heartbeats between breaths [40] and a conditional fitting algorithm to take into consideration the brief recovery intervals between inversion pulses.

Noninvasive detection and quantification of myocardial diseases involving the interstitium and the myocyte, like fibrosis, edema, and insoluble fiber deposition, are possible by myocardial T1 mapping sequence, which has high diagnostic accuracy (up to 92 % for myocardial T1 cutoff of 1020 ms) [23] and can evaluate the myocardial T1 relaxation time without gadolinium administration. T1 mapping is particularly useful for diagnosing and monitoring conditions like myocarditis, cardiomyopathies, and infiltrative diseases.

Native T1 values are influenced by the field strength (higher values at 3 T vs. 1.5 T), pulse sequence (ShMOLLI underestimate T1), and mean hazard ratio (HR), and therefore, they are specific to the local set-up, and they are also MRI vendors specific [24].

MOLLI-T1 normal range values are significantly higher at 3.0 T than 1.5 T (1149 ± 58 ms vs. 978 ± 36 ms; *p* < 0.001).

Values of 950 ± 21 (1.5 T) and 1286 ± 59 (3 T) have been reported for amyloid involvement. However, high T1 and ECV values can be seen in acute MI as well [25]

ECV is a quantitative parameter derived from T1 mapping data. It provides an estimation of the fraction of the myocardial tissue volume that is made up of extracellular space, which includes interstitial fluid and fibrotic tissue. ECV is calculated by comparing the T1 relaxation time of myocardial tissue before and after the administration of a gadolinium-based contrast agent [26]. In contrast to other cardiac disorders, CA is characterized by the highest native T1 and ECV values [25], and in confirmed cases of CA, reported cardiac ECV values range from 44% to 61%, while in healthy volunteers, they range between 22% and 27% [27].

Compared to other cardiomyopathies and acute myocarditis, cardiac amyloid has a higher native T1 and ECV (ECV 46.6 ± 7.0%) because of the extensive and significant extracellular infiltration [27].

The ECV value is higher in ATTR, meaning that the amount of amyloid is proportionally higher in ATTR than in AL hearts. The native T1 is, however, lower. The differences in ECV and T1 between ATTR and AL amyloidosis suggest a potential variation in myocyte response and provide a novel perspective on the pathogenesis of cardiac amyloidosis [28].

According to the diagnostic algorithm proposed by the recent guidelines, CMRI is indicated for diagnosing CA in case of discordant findings between clinical findings and first-tier diagnostic investigations [2].

**Fig. 1.**
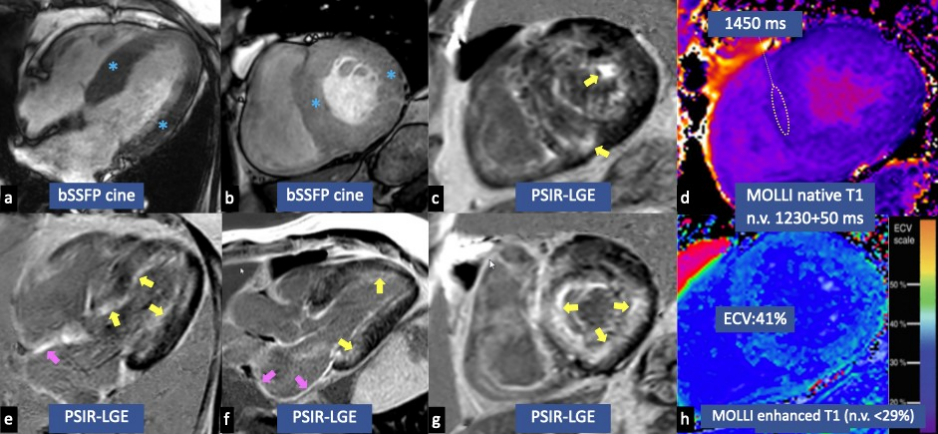
**Cardiac magnetic resonance imaging (CMRI) findings of cardiac amyloidosis (CA). **CMRI shows a case of CA characterized by the presence of concentric left ventricular hypertrophy (blue asterisks in a,b), circumferential subendocardial late gadolinium enhancement (LGE) (yellow arrows in c,e,f,g), atrial LGE (pink arrows in e,f), very high global native myocardial T1 values (d) and high extracellular volume (ECV) (h). bSSFP, balanced steady state free precession; PSIR, phase sensitive inversion recovery; MOLLI, modified look-locker inversion recovery.

**Fig. 2.**
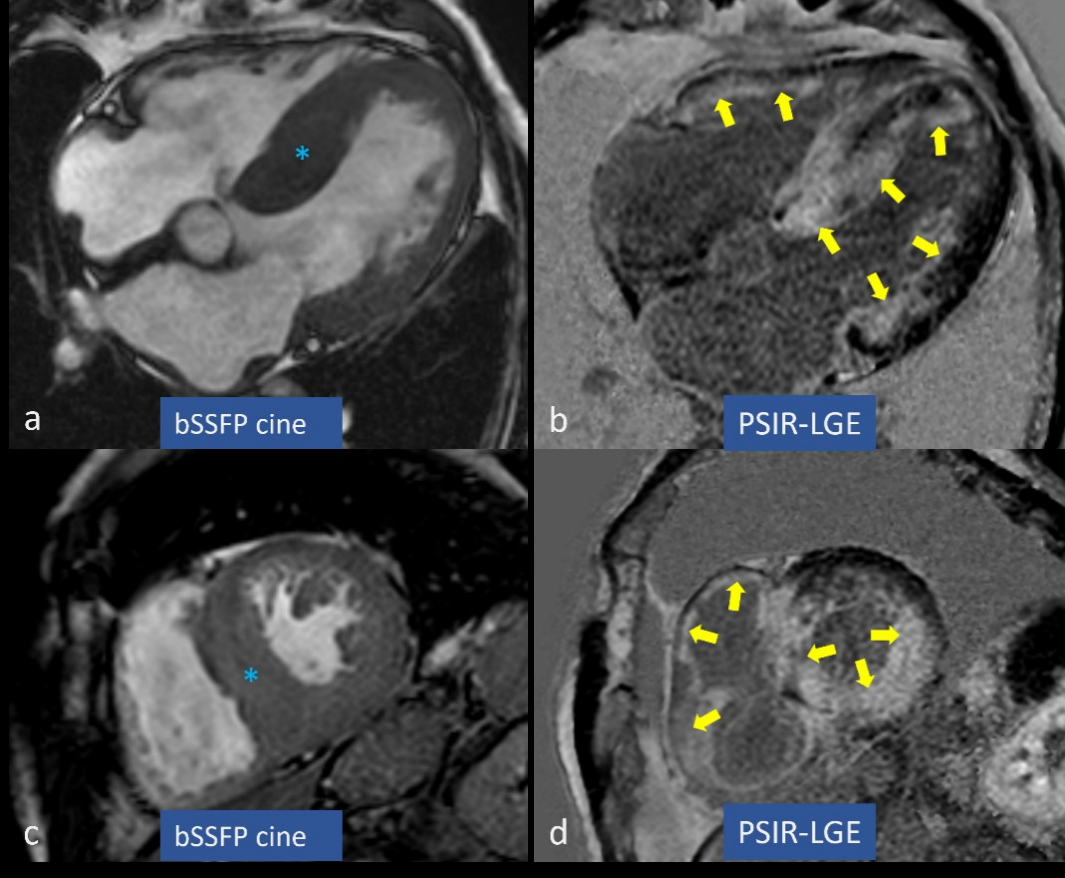
**Left ventricular hypertrophy and late gadolinium enhancement (LGE) in cardiac amyloidosis (CA). **Concentric left ventricular hypertrophy is marked by blue asterisks in (a,c), while circumferential subendocardial and partially transmural LGE is highlighted by yellow arrows in (b,d). bSSFP, balanced steady state free precession; PSIR, phase sensitive inversion recovery.

Typical MRI features of CA are: concentric left ventricle hypertrophy (Figs. 1a,b,2a,c) often associated with atrial walls thickening (Fig. 1a); restrictive configuration of the heart characterized by large atria, small ventricles, and reduced longitudinal shortening of the LV; global, circumferential subendocardial LGE (Figs. 1c,e,f,g,2b,d) defined “subendocardial tramline” or “zebra-pattern” with a non-coronary distribution; blurry, inhomogeneous suppression of myocardial signal and dark blood pool on LGE; atrial LGE (Fig. 1e,f); very high global native myocardial T1 values (Fig. 1d) and high ECV (Fig. 1h); pleural and pericardial effusions [29].

CMRI can assess well both AL and ATTR-CA. The AL subtype is characterized by a global subendocardial LGE, while the ATTR amyloidosis shows a transmural LGE pattern that spares the apex (base-apex gradient) and frequent RV involvement. However, these findings have low specificity in differentiating the two subtypes; therefore, they are not indicated routinely for distinguishing the two forms of disease [30].

### 3.2 Strain CMR Analysis 

CMRI has been revealed to be useful in assessing the strain of cardiac chambers.

To assess cardiac muscle motion and deformation, numerous CMR approaches have been developed recently [31]. Tagging and feature tracking are advanced techniques used in cardiac MRI to assess the function of the heart. Tagging involves applying a series of magnetic markers or tags to the myocardium. These tags can be visualized during the MRI scan and provide information about the motion and deformation of the heart during the cardiac cycle [32].

By tracking the displacement of the tags during the cardiac cycle, it is possible to quantify various parameters related to heart function, such as strain (the deformation of the heart muscle), strain rate (the speed of deformation), and myocardial velocity.

Feature tracking is a post-processing technique. It is an optical technique that relies on identifying particular elements in the already obtained long-axis and short-axis cine steady-state free precession (SSFP) cine images and then following them in succeeding images to produce a sequence [33]. This method can be used to calculate the displacement of myocardial segments.

It is based on creating tiny square windows that are centered on a feature on a first image and looking for the closest-matching greyscale pattern on a follow-up image [34]. Maximum likelihood techniques are used to locate the anatomical components in two regions of interest across two frames that make up the CMR-feature tracking (FT) features. These anatomical components differ along the cavity-myocardial tissue border. The automatic border tracking feature of CMR-FT software begins after manually drawing the boundaries of the endocardium and the epicardium. It evaluates radial and circumferential strains from the short-axis SSFP cine images and longitudinal strain from long-axis SSFP cine images.

The main limitation of FT is represented by through-plane motion artefacts [35].

These techniques are useful in assessing myocardial function in patients with CA, as the disease can lead to abnormal myocardial mechanics. By analyzing the patterns of deformation and motion, tagging and feature tracking can provide valuable information for diagnosing and monitoring CA, as well as guiding treatment decisions. In one study, authors evaluated 61 patients with systemic amyloidosis undergoing 3.0-T CMR with CMR tagging and LGE imaging. They found that among 48 LGE-positive and 13 LGE-negative patients, the peak circumferential strain (CS) was significantly lower in the LGE-positive than in the LGE-negative amyloidosis group (–9.5 ± 2.3 vs. –13.3 ± 1.4%, *p* < 0.01). The authors also found that CS correlated well with clinical biomarkers, such as BNP N-terminal fragments (NT-proBNP) and cardiac troponins, and the severity of CA. The diagnostic accuracy for identifying LGE-positive amyloidosis patients using CS parameters was high, with a sensitivity, specificity, and overall accuracy, respectively of 93.8%, 76.9%, and 90.2% [41]. Pandey *et al*. [42] have further demonstrated a relevant decrease of global ventricular strain (circumferential, radial, longitudinal), especially in the basal level, which had high diagnostic accuracy for differentiating amyloidosis-positive versus negative patients (sensitivity and specificity of 82.5% and 82.9%, respectively).

In another study, a reduction of basal, mid-peak radial, and peak circumferential strains has also been associated with higher levels of basal extracellular volume (ECV) [43]. The usefulness and accuracy of strain imaging in assessing CA were confirmed by the recent findings of Reddy *et al*. [44], which found a similar reduction of the radial, global, and longitudinal strain of the left ventricle when echocardiographic were compared with CMRI findings.

Also, the evaluation of left atrial strain (LAS) by CMRI has a high detection rate of amyloid deposits in the left atrium. In one study, reservoir LAS, booster LAS, and left atrial strain rate (LASR) were all reduced in biopsy-proven cases of CA when they were compared to the findings obtained in healthy individuals (*p* < 0.001) [28], and early and precontraction atrial longitudinal strain was reduced in the LGE-positive amyloidosis patients if compared to the negative control group [45].

Strain imaging by CMRI is also useful to differentiate CA from other conditions, such as hypertensive heart disease (HHD). Zhang *et al*. [46] evaluated myocardial strain by feature tracking technique in 25 patients with CA, 30 sex- and age-matched patients with HHD, and 20 healthy controls.

All patients (CA and HHD) exhibited an impairment of left ventricular strain (LVS), but CA patients had a most pronounced gradient of radial and longitudinal strain from the basal to the apical myocardium, and the apical sparing ratio was significantly higher in CA versus the other two groups of patients (CA: 0.91 ± 0.02; HHD: 0.72 ± 0.02; controls: 0.56 ± 0.01, all *p* < 0.001) [47].

Strain CMRI analysis can also differentiate CA from several other disorders, such as the Anderson-Fabry disease using feature-tracking software (Velocity Vector Imaging) [46], constrictive pericarditis using feature-tracking [48], and hypertrophic cardiomyopathy (HCM) [49].

Also, the study of the right ventricular strain with the MRI feature tracking can be helpful to differentiate CA from HCM [50]. In one study, the CMR feature tracking of 43 CA and 20 HCM patients showed different global RV longitudinal strain (–16.5 ± 3.9% vs. –21.3 ± 6.7%, *p* = 0.032; –19.8 ± 4.8% of controls), radial strain (–11.7 ± 5.3% vs. –16.5 ± 7.1%, *p* < 0.001; –19.7 ± 8.5% of controls) and circumferential strain (–7.6 ± 4.0% vs. –9.4 ± 4.4%, *p* = 0.015; –11.7 ± 3.0% of controls), compared with those measured in healthy controls. In the same study, the two CA groups (ATTR-CA and AL-CA) had similar strain values, while the right ventricular ejection fraction (RV-EF) was significantly different between CA and HCM groups (*p* = 0.017) [50].

In another study, the reservoir (R), conduit, booster rate atrial strain (RAS), and rate atrial strain rate (RASR) differ significantly between CA and HCM groups (R: 10.6 ± 14.3% vs. R: 33.5 ± 16.3%, *p* < 0.001) and also with the control group (R: 44.6 ± 15.7%, *p* < 0.001). Moreover, an impaired reservoir RAS and RASR were higher in patients with AF as compared to the individuals with sinus rhythm (SR) (6.1 vs. 14, *p* = 0.007 for RAS and 0.4 vs. 0.9, *p* = 0.008 for RASR) [51].

Eckstein *et al*. [52] have also found a higher impairment of both left and right ventricular EF (*p* < 0.001) and also of left and right atrial reservoir strain of CA versus HCM patients and controls (RA; HCM: 33.5 ± 16.3% vs. CA: 10.6% (5.6; 19.9), *p* < 0.001; LA (four chambers); HCM: 14.7 ± 7.1% vs. CA: 7.0% (4.5; 11.1), *p* < 0.001). The main echocardiographic and CMRI findings are described in Table 1.

### 3.3 The Prognostic Role of CMRI

Beyond diagnostic applications, CMRI makes a significant contribution in terms of prognosis and prediction of adverse events.

The presence of LGE is associated with cardiac arrhythmias, microcirculatory dysfunction, myocardial dysfunction, progression to end-stage heart failure, and a worse prognosis [53, 54, 55, 56].

A systematic review of 7 studies enrolling 425 patients has found that patients with CA and LGE have a significantly higher mortality rate than those without (pooled odds ratio (OR): 4.96; 95% confidence interval (CI): 1.90 to 12.93; *p* = 0.001) [57].

In histologically confirmed AL-CA, the presence of diffuse LGE at CMRI was also associated with the highest mortality for all causes (HR: 2.93; *p* < 0.001), confirming a good predictive value in comparison with cardiac biomarkers [58]. It has also been found that LGE has a high prognostic value for predicting the severity of heart failure compared to the brain natriuretic peptide (BNP) [59].

Transmural LGE is a strong predictive factor of death (HR: 5.4; 95% CI: 2.1–13.7; *p* < 0.0001), also after adjustment for N-terminal pro-brain natriuretic peptide, EF, stroke volume index, and left ventricular mass index [60].

Survival rate was lower in AL-CA patients with positive versus those with negative CMRI (28%, 14%, and 14% vs. 84%, 77%, and 45% at 1, 2, and 5 years, respectively, *p* = 0.002). Among CMRI-positive patients, the presence of LGE, biventricular hypertrophy, and pericardial effusion were the best predictors of a decreased survival rate [61].

Modern imaging techniques (T1 and ECV quantification in CMR) allow clinicians to understand better how patients respond to treatment and to personalize treatment plans for each patient [62, 63].

In another study of AL-CA patients, an ECV ≥44.0% and global LGE were independent risk factors of mortality when compared to the other clinical and instrumental findings (respectively, HR of 7.249, 95% CI: 1.751–13.179, *p* = 0.002 and HR of 4.804, 95% CI: 1.971–12.926, *p* = 0.001) [64]. Furthermore, Li X *et al*. [63] have found that LGE and GLS were independent predictors of mortality (respectively for left ventricle, HR: 2.44, 95% CI: 1.10–5.45, *p* = 0.029 and HR 1.13 per 1% absolute decrease, 95% CI: 1.02–1.25, *p* = 0.025 and for right ventricle, HR: 4.07, 95% CI: 1.09–15.24, *p* = 0.037 and HR 1.10 per 1% absolute decrease, 95% CI: 1.00–1.21, *p* = 0.047) [63]. CA patients also exhibit lower values of right ventricular global radial peak strain (RV-GRPS), global circumferential peak strain (GCPS), and global longitudinal peak strain (GLPS) than controls (respectively 20.3 ± 2.12 vs. 31.31 ± 7.61, –2.12 ± 0.88 vs. –13.71 ± 2.53 and –5.33 ± 0.64 vs. –14.239 ± 2.99). RV-GRPS was the strongest predictor of mortality in these patients (HR: 0.93, 95% CI: 0.88–0.98, *p* = 0.007) [64].

The finding of a reduced longitudinal atrial strain and myocardial contraction factor has been associated with high mortality hazard and need for heart transplantation (HR respectively of 1.05 and 0.96, all *p* < 0.001) [65].

Some authors have classified the burden load of amyloid fibrils according to the CMRI detection of LGE and ECV. The patients with the highest amyloid burden load had a significant reduction of left atrial reservoir strain (LARS), conduit, booster strains, and ASR, and according to Kaplan-Meier analyses, the presence of low LARS values (<8.6%) were strongly correlated with the highest risk of death [66].

### 3.4 Costs and Benefits of CMRI

In recent years, the interest of clinicians in the field of CA has gradually increased with the improvement of imaging techniques such as CMRI.

The use of CMRI is, however, limited by various factors, high costs and the need of skilled operators, and CMRI may be contra-indicated in the occasional non-MRI conditional devices [67, 68, 69]. Despite these limitations, CMRI can sometimes detect early findings of CA and is useful also to drive the treatment and monitor the response to the therapy [67].

Among patients suspected of having or at risk for AL-CA, parametric CMR measurements (T1/ECV and ECV) outperformed echocardiographic measures such as LVEF and GLS and in predicting both mortality and heart failure hospitalizations [67, 68].

ECV changes are independently associated with the prognosis of patients with light chain amyloidosis, underscoring the unique role of MRI in assessing treatment response [67].

The development of specific therapeutic strategies, such as transthyretin stabilizers, which has been associated with an improvement of overall survival, quality of life and with a reduction in hospitalization time [67], has highlighted how an early diagnosis a prompt treatment could change the natural history of the disease in some cases, reaching a positive cost-benefit ratio in these patients. Clinicians should be encouraged, therefore, to perform a screening of suspected cases and to refer them to specific referral amyloid centers [68]. It has been found that sequential tests involving 99-m technetium pyrophosphate and cardiac magnetic resonance imaging may be cost-effective ($150,000/quality-adjusted life year) and, therefore, can be useful for detecting early cases of CA and improving the outcomes [69, 70].

## 4. Conclusions

Echocardiography undoubtedly plays a crucial role in the assessment of CA patients. However, the specificity of echocardiographic findings may be limited, with early, nuanced features of the disease often only discernible by highly experienced practitioners and often necessitating additional confirmatory tests. CMR imaging ascends as a more accurate method, not only for the detection of suspected CA cases and differential diagnosis of LVH phenotypes but also as a robust tool for monitoring disease progression and response to treatment. The recent integration of CMR into guideline-directed diagnostic algorithms has significantly amplified clinical awareness of potential CA cases, simultaneously reducing reliance on invasive endomyocardial biopsies. Particularly with its advanced tissue characterization capabilities, CMR contributes valuable insights that augment the precision for the timely detection of CA.

The application of a validated diagnostic algorithms incorporating sequential non-invasive cardiovascular imaging modalities has proven effective in securing a diagnosis in a considerable proportion of cases. Extracardiac biopsies may serve as a valuable tool to elucidate additional cases, while endomyocardial biopsies can be reserved for a minority of cases when other diagnostic efforts remain inconclusive. The early identification of CA cases, thereby enabling the initiation of targeted treatments, can substantially impact patient survival rates, hospital stay duration, and overall quality of life. Therefore, it is critical for clinicians to expedite referrals of suspected cases to specialized amyloid centers, ensuring patients receive the most suitable diagnostic pathway and comprehensive care.
